# Supplementary material for: Study protocol for the Alzheimer and music therapy study: An RCT to compare the efficacy of music therapy and physical activity on brain plasticity, depressive symptoms, and cognitive decline, in a population with and at risk for Alzheimer’s disease
Source: PLoS One. 2022 Jun 30;17(6):e0270682. doi: 10.1371/journal.pone.0270682 (PMC9246122; doi:10.1371/journal.pone.0270682)
Supplement: S1 Table — The different versions and amendments to the Study Protocol. (DOCX) [file pone.0270682.s001.docx]

**S1 Table.** *Changes to the Study Protocol*

| Version | Date | Domain | Change | Rationale |
| --- | --- | --- | --- | --- |
| 1.2 | 29.10.18 | Inclusion Criteria | Mild Cognitive Impairment (MCI) | Recommendations from a retrospective feasibility assessment led to extension of inclusion criteria. |
| 1.2 | 29.10.18 | Measurements | Free and Cued Selective Reminding Test (FCSRT) | FCSRT was included as it is sensitive to preclinical and prodromal AD. |
| 1.3 | 04.01.19 | Inclusion Criteria | Memory complaints without a clinical diagnosis | Recommendations from a retrospective feasibility assessment led to extension of inclusion criteria. |
| 1.3 | 04.01.19 | Measurements | Subjective Cognitive Decline-Questionnaire | Measure subjective memory complaints. |
| 1.4 | 15.01.19 | Measurements | Six-Minute Walk Test, Grip Strength Test, International Physical Activity Questionnaire short version | Include physical outcome measures. |
| 1.5 | 28.01.19 | Recruitment | Healthy Control subjects to undergo MRI measurements | To be able to calculate BrainAGE. |
| 1.6 | 15.01.20 | Measurements | Dispositional Resilience Scale-15 and Goldsmiths Music Sophistication Index | To be able to describe the sample’s baseline resilience and musical background. |
