# Supplementary material for: Study protocol for the Alzheimer and music therapy study: An RCT to compare the efficacy of music therapy and physical activity on brain plasticity, depressive symptoms, and cognitive decline, in a population with and at risk for Alzheimer’s disease
Source: PLoS One. 2022 Jun 30;17(6):e0270682. doi: 10.1371/journal.pone.0270682 (PMC9246122; doi:10.1371/journal.pone.0270682)
Supplement: S1 File — The original protocol approved by The Regional Committee for Medical and Health Research Ethics. (PDF) [file pone.0270682.s003.pdf]

ALzheimer and MUsic THerapy: Randomised trial of Singing Lessons versus Exercise or No Treatment on Brain Age and Depression Symptoms in People with Alzheimer Disease (ALMUTH)

Administrative Information

Project Manager

Stefan Koelsch

Professor
Institutt for biologisk og medisinsk psykologi
Det psykologiske fakultet
Universitet i Bergen

Stefan.Koelsch@uib.no
+47 55 58 62 31

Jonas Lies vei 91
Postboks 7807
5020 BERGEN

Co-Authors

Ulvhild Færøvik, Christian Gold

Project Title

Scientific title: ALzheimer and MUsic THerapy: Randomised trial of Singing Lessons versus Exercise or No Treatment on Brain Age and Depression Symptoms in People with Alzheimer Disease (ALMUTH)
Short title: ALzheimer and MUsic THerapy: Trial of Singing Lessons for People with Alzheimer
Acronym: ALMUTH, which stands for ALzheimer and MUsic THerapy.

Trial Registration

The project was released to ClinicalTrials.gov for review on February 16, 2018.

Funding

The project is funded by Norsk Forskningsråd and the Institute of Biological and Medical Psychology (IBMP) at the University of Bergen (UiB). The authors declare that they have no conflicts of interest. The funder will not have any role in the design or conduct of the study, in the writing of the report, or in the decision to submit the report for publication.

Roles and Responsibility

Professor Stefan Koelsch is the project leader and responsible for the execution and the overseeing of the project.

Sebastian Jentschke is a researcher on the project and in charge of analysis and technical administrations.

Ulvhild Færøvik is a PhD student on the project and is in charge of coordination, data gathering, applications, research activity and material.

Kathrine Dahle is affiliated with the project on a 30 % basis and in charge of recruiting participants through Bergen municipality and research activity.

Professor Christian Gold is affiliated with the project 20 % and in charge of randomisation of participants, applications and research activity.

Anne Henriksen, Frøydis Hausmann and Tobba Therkildsen Sudmann are all affiliated with the project as councilors on the quality and guidance of the exercise interventions. The three of them work at Høgskulen på Vestlandet (Campus Bergen) and teach physical health.

There will be two additional PhD students affiliated with this project full time. None of these have been hired yet, but the process has started. There is also a announcement for a position as a researcher or Post Doc.
Contact information for the researchers can be found here:
http://www.uib.no/en/brainandmusic/112850/alzheimer-and-music

Abstract

Music has powerful effects on memory in patients with neurodegenerative diseases. However, although there is anecdotal evidence for beneficial effects of active music interventions in patients with Alzheimer's disease (AD), there is lack of high-quality research investigating this issue, and the cognitive, emotional, and social factors that contribute to potentially beneficial effects of music making in AD patients are largely unknown. In a randomised controlled intervention trial, a cohort of AD patients will undergo twelve months of music lessons specifically tailored for AD patients. Another group of AD patients will undergo twelve months of exercise lessons and a third group of AD patients will be in a passive control group. Structural and functional magnetic resonance imaging (MRI) will be used to determine changes in brain age (as compared to two control groups), and voxel-based morphometry will be computed to determine contributions of different factors of the music intervention (cognitive, emotional, and social) to plastic changes of brain morphology, and a potential deceleration of brain atrophy. In addition, quality of life of patients. In cooperation with the Bergen municipality, and a strong network of national and international partners, music therapy will be implemented, involving training of music therapists, and communication of results to patients, patient groups, and therapists.

Introduction

Background

Alzheimer's disease (AD) (Selkoe, 2001) is a neurodegenerative disease where patients experience a worsening of episodic and semantic memory, cognitive functions and language. Behavior is also affected by the disease as a result of loss of norm understanding, paranoia and delusions. Selkoe and Dennis (2001) points out that AD symptoms may be improved with medicine, but there is no cure for AD.

According to figures from the Norwegian Institute a Public Health Report from 2014, every fifth person in Norway will develop dementia during their lifetime. Furthermore, in the 2014 report, 42 000 people in Norway will get AD, which is the most common form of dementia.

A literature study (Hulme, Wright, Crocker, Oluboyede, & House, 2010) examined 33 literature reviews on non-medical treatments of dementia. In their survey they identified music or music therapy as the strongest, most efficient non-drug treatment of dementia. The authors further pointed out that music was efficient for calming people with dementia and hindered wandering. Exercise was identified as the second most effective treatment of dementia in this literature review. The studies all tested dementia patients in later stages of the disease (not persons who stilled lived at home).

Singing and music interventions can have several benefits in themselves. One study (Vickhoff et al., 2013) found that singing had a positive effect on cardiovascular activity. Another study (Tarr, Launay, & Dunbar, 2014) found that singing has a strong effect on social bonding. A third study (Osman, Tischler, & Schneider, 2016) examined a singing group intervention for people with dementia and their caregivers, and found that patients experienced improvement in memory, both patients and carriers had betterment in their mood and relationship. Dassa and Amir (2014) found that music therapy with songs from the past of AD patients provided them with access to memories that led to spontaneous conversations. Patients also reported that they had a positive sense of well-being, belonging and a sense of achievement after music lessons. However, the above-mentioned studies all looked at people in late stages of AD.

A study (Hsieh, Hornberger, Piguet, & Hodges, 2011) found that despite severe impairment of episodic (and moderate impairment of semantic) memory, some patients with AD have nearly-preserved memory of musical information. In a study by Vanstone et al. (2012) five out of twelve patients with moderate or severe AD performed as well as the healthy control group in tests such as distinguishing familiar from novel melodies, identifying distortions in melodies, and singing familiar tunes. Another study (Cuddy et al., 2012) showed that long-term familiarity for melody was preserved across levels of AD (mild, moderate, and severe), and that the ability to sing a melody when prompted by its lyrics was retained at the mild stage and by a few individuals even through the severe stages of AD. Thus, musical semantic memory may be spared through the mild and moderate stages of AD and may be preserved even in some individuals at the late stage. A case study (Moussard, Bigand, Belleville, & Peretz, 2012) with a mild AD patient reported that learning of sung lyrics (even on unfamiliar melodies) led to better retention of words in AD patients. This finding is consistent with evidence (El Haj, Clément, Fasotti, & Allain, 2013) suggesting that relatively brief exposure of AD patients to music (such as listening to music for a few minutes) has effects on language production in terms of fewer empty words, higher grammatical complexity and pro-positional density. In addition, brief music exposure has beneficial effects on episodic memory retrieval (Foster & Valentine, 2001; Irish et al., 2006) even if the music is unrelated to the recalled autobiographical event (El Haj, Postal, & Allain, 2012). One of the reasons why musical interventions work well for older patients with AD is that musical memory is relatively intact (Jacobsen et al., 2015). Another study (Oostendorp & Montel, 2014) found that older AD patients could learn and remember new information with singing, despite suffering from severe memory loss. The authors suggested that future research should look at whether singing can prevent memory loss for individuals with AD in earlier stages of the disease.

The findings above indicate that memory for musical information is less impaired than episodic and semantic memory in AD patients (whether musical memory is represented as music-semantic memory, or procedural memory, or both). Moreover, the findings suggest that activations of representations of musical memory can lead to benefits in the domains of episodic and semantic memory retrieval, as well as in the domain of language production. The notion of a separate musical memory that can exert positive effects on episodic and semantic memory impairment is consistent with a recent case study (Finke, Esfahani, & Ploner, 2012) of a 68-year-old professional cellist who developed severe amnesia following encephalitis: Despite severe semantic and episodic memory impairments, this patient performed like healthy musicians in various tests of recognition memory for music. Moreover, the patient was strikingly unimpaired in the learning and retention of new musical information (despite severe impairment in learning, e.g., new faces). The authors concluded that "learning and retention of musical information depends on brain networks distinct from those involved in other types of episodic and semantic memory", and that "learning and memory of complex musical information constitute an island of intact cognition within a severe amnesic syndrome." (Finke et al., 2012).

Music and emotion in AD. In addition to memory- and language-related phenomena, music can aid to reduce anxiety and depression of AD patients. For example, a randomised controlled trial (Guetin et al., 2009) suggests that patients with mild to moderate AD show a reduction of anxiety and depression scores over the course of weekly sessions of receptive music therapy (compared to a reading group). This finding is consistent with several recent meta-analyses on effects of music therapy in AD patients. However, all of these meta-analyses (McDermott, Crellin, Ridder, & Orrell, 2013; Ueda, Suzukamo, Sato, & Izumi, 2013; Vink, Bruinsma, & Scholten, 2003; Wall & Duffy, 2010) also noted that the methodological quality of studies was low. For example, Ueda et al. (2013) concluded that "it seemed that depression and anxiety were reduced in the patients with the Alzheimer's type of dementia, but the number of studies was insufficient to establish an effect on this type of dementia". Similarly, McDermott et al. (2013) concluded that "Evidence for short-term improvement in mood and reduction in behavioural disturbance was consistent, but there were no high-quality longitudinal studies that demonstrated long-term benefits of music therapy." Wall & Duffy (2010) reported positive increase in participants' mood and socialization skills across studies, but also noted that "methodological limitations were apparent throughout each of the studies reviewed", and recommended "the undertaking of further research to explore the effects of music therapy on the behaviour and wellbeing of older people with dementia." For the present study it is worth noting that no previous study investigated AD patients who had already learned an instrument (e.g., during childhood and adolescence); however, active music making in the form of singing has been applied in a few studies, and the meta-analysis by McDermott et al. (2013) found that "singing featured as an important medium for change." A meta-analysis (Fusar-Poli, Bieleninik, Brondino, Chen, & Gold, 2017) found that music therapy is important as a complimentary treatment for older adults with dementia; specifically, the review found that active (music making) but not receptive methods (music listening) had a beneficial effect on cognitive abilities; subgroup analysis found evidence of a beneficial effect of active MT on global cognition ($SMD = 0.29$, 95% CI 0.02 to 0.57, $p = 0.04$). Thus, there are hints that active music making (as planned in the present study) may be more beneficial than simply listening to music.

Interaction of memory functions with music-evoked emotions is plausible due to the co-localization of memory functions and emotion in the hippocampus. In a recent review (Koelsch, 2014) the applicant showed that music-evoked emotions involve the hippocampal formation across several studies (beyond neural systems involved in reward and pleasure). Moreover, recent research (Jacobsen et al., 2015; Pantev & Herholz, 2011) suggests that brain areas underlying musical memory are among the last to show atrophy in AD patients.

Theoretical models for beneficial effects of music in AD. There is also a paucity of theoretical models underlying effects of music in AD patients. Thus, many previous studies (McDermott et al., 2013) suffer from weak motivation for using music and lacing definitions of outcome measures. Moreover, due to the lack of theoretical models, there is a lack of systematic evidence for different factors that might contribute to beneficial effects, including the well-being of dementia patients. The present project aims at identifying such factors (see next section), thus also establishing a new theoretical basis for MT research with dementia patients.

The strong effects of music, in particular music making, on brain plasticity have been shown in a plethora of neuroscientific studies with both healthy individuals (Jäncke, 2009; Münte, Altenmüller, & Jäncke, 2002) and patients (Särkämö et al., 2008). A study used functional magnetic resonance imaging technique (fMRI) (Satoh et al., 2015) and AD patients underwent music therapy sessions once a week for six months. After six months, patients had increased activity in right angular gyrus and left lingual gyrus and they did better on cognitive processing exercises. In England a study (Skingley, Martin, & Clift, 2016) where elderly people sing in groups together, participants experienced improved well-being and health.

Research has shown that it is possible to prevent falls by exercising. An alternative intervention, which is helpful for the users, is exercise. There has been research on dementia and effects of exercise (Hulme et al., 2010) where training were considered an effective non-medical treatment compared to other non-medical treatments. In Bergen Municipality's report "Safe on Two Legs" Bergen Kommune (2010) reported that balance training and strength have the best results for preventing falls. In the report, more studies (Campbell, Robertson, Gardner, Norton, & Buchner, 1999; Helbostad, Leirfall, Moe-Nilssen, & Sletvold, 2007; Skelton, Dinan, Campbell, & Rutherford, 2005) displayed how inactive seniors who had not previously experienced falls had a good effect of one year's training. The training group reduced the number of falls by 30% compared with the control group. The effect of exercise was also greatest in the elderly with initial or established functional failure. People with Alzheimer's have functional failure of cognitive functions, languages, and the like. According to the Safe on Two Legs report (2010), muscle strength is the low-impact effect a muscle or muscle-group can develop. Muscle strength is something that decays when people get older, and the average muscle density of a person in their 60s is about 40% less than in a 20-year-old. In an article (Liu & Latham, 2009), it appears that elders that exercise strength become stronger and achieve better function in daily activities. Another study (Kalapotharakos, Michalopoulos, Tokmakidis, Godolias, & Gourgoulis, 2005) showed that two groups of elderly people who did use to exercise did better on functional tests after a training intervention. According to the Bergen Commune report, exercise for the elderly as for young people must be done 2-3 times a week with a certain intensity (8-12 repetitions and 3 sets) and be done for a minimum duration of 12 weeks to have an effect (2010).

The reason we want to do our study is the lack of good evidence-based qualitative research in the field of music therapy and neuroscience. Most of the research on AD patients is done in later stages of the disease, and it has been recommended to try and look at music intervention as a prevention of deterioration in AD. Therefore the current project will investigate the effect of music intervention on AD patients in early stages of the disease.

Specific Objectives

The purpose of the project is to improve health for patients who are in an early stage of Alzheimer's disease (AD). It is expensive for the municipality with nursing homes, and the goal of the project is to find a therapy that can keep people with AD at home as long as possible. The aim is also to increase the quality of life in AD patients. Scientifically, the purpose is to look at changes in the

mind of persons with AD, who undergo a music intervention, compared with AD patients who do not undergo a music intervention.

Research Questions

1. How does the brain change after a music intervention for patients with AD, compared to other AD patients who have not received a music intervention?

2. How does depression severity change in patients with Alzheimer's in the various intervention groups?

3. Can song training stop natural disease progress in patients with AD?

4. Is there a difference in the brain of a group that gets a music intervention compared to a group that does not receive music intervention for patients with AD?

5. How do cognitive functions like attention, language and memory change after a song intervention on patients with AD?

Trial Design and Rationale

A quantitative design has been selected for the possibility of generalisation. Functional Magnetic resonance imaging technique (fMRI), diffusion tensor imaging (DTI) and magnetic resonance data will be retrieved from patients to measure brain-plasticity before and after music intervention. Neurological tests will be conducted to measure cognitive, social and motor functions before and after the intervention. Data will be collected by competent staff. The interventions will be performed by competent staff, respectively, vocal teachers, conductors, music therapists, physiotherapists and occupational therapists.

Trial Design

This is a parallel, three-arm, single-blind (assessor-blinded) randomised controlled trial. Participants will be randomised to either a music intervention (singing lessons), a non-musical training intervention or no intervention. To ensure allocation concealment, the randomisation list will be created and kept at an external research office at Uni Research, by researchers who have no direct involvement with the clinical work.

Methods

Study Setting

Data will be collected in Bergen Norway, at the university hospital Haukeland or at the IBMP which is located behind Haukeland and is considered part of the university of Bergen, as well as part of the university hospital.

After discussions with health professionals in the Bergens area, it is already assumed that we have to recruit from day centers in and around Bergen, such as Askøy, Sotra, Øygarden, Os and others. Sogn og Fjordane is also potential place for recruitment.

Eligibility Criteria

All adults with AD, above the age of 18, of any gender, are eligible to participate. In addition to having a diagnosis of AD, participants must live at home (not in a residential aged care facility, however they may have plans to move to such a facility in the future) and be able to give informed consent for the study. They also need to be able to complete questionnaires in Norwegian, to undergo magnetic resonance scans, and to attend intervention and assessment sessions in the area of Bergen, Norway. Participants can be non-musicians, amateur/hobby musicians, or professional musicians.

If participants move to a nursing home during the intervention period, they will not be excluded. All participants randomised will be retained and analysed, according to the intention-to-treat principle (see Statistical analyses).

Exclusion Criteria

Participants with hearing impairments that cannot be mended by hearing aids will be excluded due to the importance of listening. Participants with claustrophobia and metal operated into the body, such as a pacemaker, will not be able to participate as that would make it impossible to gather fMRI data.

Interventions

All will be provided by appropriately qualified personell, but may be provided by advanced students under supervision of a qualified professional. Singing lessons will be provided once a week for a period of 12 months by a person with a qualification in music therapy, or by a person with a different relevant qualification (e.g. psychology and music teaching) under the supervision of a music therapist. Exercise will be provided by a occupational therapists or a physiotherapist or students under supervision.

Music Intervention

The music intervention consists of singing lessons and choir practice.
Singing lessons will take place once a week for each participant every week for about 12 months. The therapist will come home to the participant, or meet them at the day centre and similar places. Participants will be given CD or MP3 files (as they prefer) with audio files with warm-up exercises, play songs and the song they will learn with their voice. Self-produced music material from the institute will be given to the participants in a media format they prefer. Example material can be found here: https://soundcloud.com/ulvhild/jeg-gikk-en-tur-pa-stien
All participants in the music intervention group will meet one to two times a month to sing the songs they have learned in the individual singing lessons together in a choir. The choir will take place in at a place devised by Bergen Municipality. The intervention is standardised (see Appendix A for a detailed schedule), but therapists are free to make small changes to adapt the individual.

Exercise Intervention

The exercise group will have similar arrangements, but meet once a week for a group training session rather than individual hours once a week. At the same time they will also be followed by a personal physiotherapist or occupational therapist before the training intervention begins and after. They will also offer group tours, such as mountain hikes and the like twice a month. We will base the training offer on the program «Trygg på to bein» by Bergen municipality (2010) intended for the elderly. The training will take place outdoors and at Bergen Municipality owned house. The intervention is quite standardised, but therapists are free to make small changes to adapt the

individual. Different from “safe on two legs”, this intervention will not use music during training sessions (see Appendix B). It will also be tailored to enable participation of all included participants.

Control Intervention

The control group will continue on medications and activities that they usually have. They will not be asked to quit training or music if they are currently participating in such activities.

Assessment of Attendance and Fidelity

Participation in the program will be documented by the therapists in charge of the intervention by simply marking if the participants attend the intervention. 80 % participation is required for inclusion in analysis.

Mid intervention we will also interview participants about how they like their therapist and the therapy, and if they are doing the interventions on their own at home. However, there will be no strict measures of whether they do the intervention on their own. This is encouraged, but not a must.

There will be a midway evaluation of therapists for both the active interventions. Here we will inquire if they overlapped in any way. Both will be asked the same questions:

«Did you use music?», «Did you use exercise?»

Then we will ask the participants the same questions about the therapy sessions. This includes double self-reports from therapists and participants. In addition we will ask participants for their compliance, or how much they practiced on their own.

Outcomes

Primary Outcomes

The study will have two primary outcomes, one neurophysiological and one behavioural.

1.) Brain age: As the primary scientific goal is to examine brain plasticity, specifically changes in the brain following the music intervention compared to the control group. We will use the Especially interesting are the hippocampus and frontal lobes. program Brain Age Gap Estimation (BrainAGE) to estimate brain age, based on structured T1-weighted MRI recordings (Franke, Ziegler, Klöppel, Gaser, & Initiative, 2010) taken with a 3T scanner. BrainAGE estimates the deviation in months of a given brain from the brain age of "healthy" people of the same age. This is related to AD and may reflect an effect of the intervention. MR scans will be taken before randomisation and after 12 months. In addition to brain age, the MR scans will also be used to explore regions of interest, especially the hippocampus and frontal lobes.

2.) Depression: Our primary behaviour goal is to examine change in depression, over 12 months. We expect that before the intervention, depression will be higher for all, while after intervention it will be lower for people both in training and music interventions, but unchanged for the control group. To measure the depression of people with AD, we will use the Geriatric depression scale (GDS) which is a single target to look at depression.

Secondary Outcomes

Secondary outcomes will include behavioural changes in the life of people with AD and the changes that occur in the brain. For secondary outcomes the music group will be compared to the training group and both will be compared to the control group on all measures. The following is a list of secondary outcomes and which tests will be used to investigate them.

Secondary Behavioural Outcomes

Title: Living independently at home
Description: A measure of when people with AD move to a nursing home
Time frame: 12 months (or longer)
Test: After 12 months of intervention we will ask our participants if they are still living at home or at a nursing home. We will also follow up with them after two and a half years and after five years to ask the same question. This is to get a deeper understanding of the longterm effects that music or exercise intervention may have on people with AD.

Title: Geriatric Depression Scale (GDS)
Description: Total score changes in the GDS which is a self-reported scale
Time frame: Testing before randomisation and after 12 months of intervention.
Test: GDS is a recognised test that is often used in psychological contexts and in research to gain and understanding of participants depressive symptoms or if they have depression. Depression is very common among the elderly. The GDS total score is the sum of 15 items, each of which is answered as yes (1) or no (0), the total score can range from 0 to 15. Higher scores indicates more severe depression symptoms. A score of > 11 indicates depression, with a sensitivity of 84 % and specificity of 95 %. However, for patients diagnosed with dementia (Burke, Houston, Boust, & Roccaforte, 1989), sensitivity and specificity has a somewhat lower cut-off score, which means that the test can only be used on people with light dementia, such as AD patients in early stages of the disease.

Title: Language abilities
Description: Word Learning Test
Time frame: Testing before randomisation and after 12 months of intervention.
Test: Language is one of the functions people with AD lose, and who often get worse with the disease. We want to see if our participants experience a recovery or not a deterioration through the intervention. Of course, this can also contribute to the effect of socialisation on a weekly basis, and that language is better taken care of by oral and interpersonal interactions. The reason we want to look at several parameters is to get an overview of what may be the different causes of change in the intervention.

Title: Sensor-motor functions
Description: Total score of the finger tapping test (FTT)
Time frame: Testing before randomisation and after 12 months of intervention.
Test: Sensor motor functions can be modified by intervention simply by stimulating work memory or focusing attention on participants during certain periods. Here we want to get a holistic impression of what is being changed and checking for other factors.

Title: Living independently with Alzheimer
Description: Alzheimer Daily Living Assessment (P-IADL) and total score of the Instrumental Activities of Daily Living (I-ADL).
Time frame: Testing before randomisation and after 12 months of intervention.
Test: We want participants who are in an early stage of Alzheimer's disease because we believe that music therapy can have a strong effect on those who can prevent the disease from developing. Therefore, we want to test the course of illness before and after interventions. Each of the eight

items is scored from one to three, four or five, for a total score ranging from 0 to 31. 0 is a none score, as it means not able to answer the questions. For example if the patient does not use medication, the score is 0, because there is no use to know if they can take it themselves. Low scores indicates high instrumental activities of daily activities, high score indicates little instrumental activities. For the other test, the totalt score ranges from 0 to 30. Low scores indicate high independence, while high scores indicate low independence.

Title: Memory and attention
Description: Total score of Mini-Mental State Examination (MMSE)
Time frame: Testing before randomisation and after 12 months of intervention.
Test: Memory and attention are two of the things that may be marked very early in AD, or that is why it is discovered. MMSE is commonly used in many Alzheimer's research and provides an idea of disease progression, as well as the ability to remember and learn. High score, or maximum score of 30 points indicates good mental state, while low scores (minimum 0) indicate poor mental state.

Title: Cognition
Description: Stroop test (online version)
Time frame: Testing before randomisation and after 12 months of intervention.
Test: Stroop test is a difficult cognitive test that challenges language, cognition and motor functions. We would like to use this test to look at cognitive abilities in people with AD. The digital version of the test presents one colour and participants are asked to name the colour. In half the trials a word is written in the coluor participants are supposed to name. This is harder as the words are other colours, such as blue, red, yellow and green (these are also the colours participants must name correctly). Score is derived digitally based on correct trials, times and higher score for matching words and wrong colours, such as blue. High score indicates high cognitive functions.

Secondary Neuroscientific Outcomes

Title: Integrity of fibre tracts
Description: Changes in the integrity of fibre tracts can be measured by Diffusion Tensor Imaging (DTI).
Time frame: Testing before randomisation and after 12 months of intervention.
Test: DTI can be used to look at the loss of fibre rays in the hippocampus, which we assume to be stronger in the other intervention groups compared to the music group. DTI also sees several biomarkers in AS, which correlates with boiler-like functions (Nir et al., 2013).

Title: Plasticity changes in the brain's morphography
Description: Voxel-based Morphography on Structural MRI, it is possible to look at differences in hippocampus, functions in the default mode network (DMN), and how music archives the brain in the default mode network. As well as functional contexts on the groups before and after intervention.
Time frame: Testing before randomisation and after 12 months of intervention.
Test: In early stages of AD, the activity of the posterior part of DMN will begin to degenerate, while in the anterior and ventral networks, activity will increase (Damoiseaux, Prater, Miller, & Greicius, 2012). We want to watch DMN with silence and a choice of various music pieces (selected by participants; see Appendix C), and see if degeneration will slow down with music intervention.

Other Measures

Tertiary outcomes will look at the differences within groups and of participants.

Title: Musical background
Description: Profile of Perception of Music Skills (PROMS) 15 minutes versions
Time frame: Testing before randomisation
Test: PROMS investigate musical skills. People with strong musical connection may prefer music intervention, but also get more out of a music intervention. Individuals are different and normally distributed in their musical abilities; it is interesting to see if music intervention for example will help better for those with stronger musical skills than those with less. It will be important for future research and treatment to know. If a person is not musical, training might be a better intervention.

Title: Physical shape
Description: Short Physical Performance Battery (SPPB)
Time frame: Testing before randomisation
Test: The Short Physical Performance Battery is a test that is used to screen physical function among elders. The test was developed to be used on people over the age of 65 years. The test has predicational values for death and when people will have to move to a nursing home (Guralnik JM, 1994). The test evaluates balance and walking speed.

Title: Background
Description: General questionnaire about gender, music background, training habits and education
Time frame: Testing before randomisation
Test: We will ask about the general background of our participants. As gender, age and education. But we also want to ask about music backgrounds and training habits. We have used some of the parts from the Montreal Battery of Evaluation of Amusia (MBEA) that ask for music backgrounds and music experience. We also ask participants about the type of interference they prefer, not because they can choose, but then we may get a pointer about why someone will drop out of the study.

Title: Midway evaluation of therapy
Description: A questionnaire about therapy and Working Alliance Inventory, Short Revised (WAI-SR).
Time frame: after 6 months of intervention
Test: We will ask about how the therapy is working, and how much participants practice on their own. This test will be to assess fidelity of both therapists and participants. WAI-SR will be used to evaluate how well the therapist works with the participants. If the relationship is poor, changes may be done.

Sample Size

The sample size calculation was based on the main comparison, music intervention compared to standard care. The two primary outcomes are equally important.
The first primary outcome, change in brain age (Franke et al., 2010), is measured on an interval scale (months deviation from normal) over 12 months. To our knowledge, no previous studies have examined intervention effects on brain age. The second primary outcome, change in total depression symptoms, as measured by the BDI over 12 months, is also a continuous measure. A previous Cochrane review of music interventions for people with depression (van der Steen et al., 2017) found small to medium effect sizes on depression symptoms (d = 0.28). However, this new music intervention will be different from previous interventions as it will be longer, have more frequent use music lessons, a daily intervention with home based practices and a choir. Singing in a choir also enhances the social aspect of the intervention. Furthermore, the study design will be different because it will be more focused on early stage AD, which possibly excludes some of the behavioural problems in previous studies. The current study will include a more homogeneous population by only including people with AD, not all types of dementia, which may also

differentiate the findings somewhat. Finally, the main comparison in this study is music intervention versus standard care, whereas the meta-analyses in the Cochrane review included a mix of comparisons with standard care and active controls. Therefore, a medium-to-large effect size (between 0.50 and 0.80) may be realistic to expect for the current study. With an assumed medium-to-large effect size of d = 0.7, a t-test with a two-sided significance level of 2.5% will reach 80% power with 40 participants in each group. To account for possible attrition, we will aim to recruit at least 45 participants per group, for a total target sample of 135 participants across the three groups. This is seen as a minimum number, so that we will not dismiss participants if we exceed the number of 45 participants in each group.

Recruitment

Participants will be recruited through media announcements and planned visits to day centres in Bergen and the areas around. We have contacted the general medical practitioners organization and informed them about the study and they have put it into their website for doctors to read if patients ask about treatment. We have also printed and distributed our flyer with the home assistents and other therapists and day centers where the patients might go.

Randomisation and Allocation Concealment

Block randomisation with randomly varying block sizes of 3 or 6 will be used to ensure balance as well as unpredictability. The randomisation list will be computer-generated and kept concealed by a researcher who has no direct contact with the participants (Christian Gold at Uni Research). The randomisation result will be revealed to the clinical investigators only after inclusion has been confirmed (eligibility confirmed, informed consent signed, baseline testing completed). Participants will be identified through an ID number and basic demographics (age, sex, diagnostic code), and the randomisation result will be communicated in written electronic form (using university email or a dedicated server for clinical trial data).

Blinding

Blinding of participants: Before randomisation, participants will be informed of the three different interventions they may be offered (of which one is no intervention). Due to the nature of the interventions, it will not be possible to blind participants to the intervention received.
Blinding of outcome assessors: Assessment of brain imaging data (including the first primary outcome) will be blinded by ensuring that the data analyst is unaware of the intervention received. The self-report measures (including the second primary outcome) cannot be blinded. The PhD students who will be responsible for data collection and interventions will not be involved in the data analysis.

Data management

All health information will be treated with the utmost confidentiality. Participants anonymity will be secured by the code key for each participant. The code keys and patient information will be locked in the office of the project manager, in two separate cabinets. Only the data collectors will have access.

Data will be stored on computers, but they will be anonymised with the code key for each participants. They will be plottet directly into spreadsheets in Excel and SPSS from paper form and computer outputs.

Treatment of Human Biological Material

Not relevant to the current project.

Statistical Methods

All primary analysis will be conducted in the modified intention-to-treat population, i.e. all who were randomised and for whom outcome data are available will be analysed in the group to which they were randomised, regardless of whether they received the full intended intervention. All continuous variables will be screened for normality of distributions. If normality is confirmed, the three interventions will be tested in an overall test (one-way ANOVA with three levels), followed by multiplicity-adjusted post-hoc t-tests (as described in Statistical analyses below). However, only one comparison – music intervention versus standard care - is regarded as the primary comparison. Multiplicity adjustment is made for the two primary outcomes, leading to a two-sided 2.5% significance level. All other comparisons and outcomes are regarded as exploratory secondary and are not multiplicity-adjusted (i.e. will be tested on a two-sided 5% level). Additional sensitivity analyses will be conducted on the per-protocol population (by treatment received). Subgroups such as gender, education will also be investigated. Adjusted measures will be music aptitude at baseline. Working alliance will be examined as a mediator variable of later clinical outcomes.

Monitoring

A data monitoring committee will not be needed because this is a low-risk intervention. The current project is not a medical trial and music and training holds little to no risk for the participants.

Handling and Reporting of Adverse Events

Little is known on adverse effects of music and training interventions. Any adverse events will be reported. The current study asks for sensitive information such as the GDS questionnaire. Appropriate action will be taken if anyone has a score that indicates suicidality or severe depression. The GDS will only use coded ID, and be done on paper, as to minimize the risk of unwanted people getting insight. All adverse events will be noted and reported, including whether the event was serious; whether it was assumed related or unrelated to the intervention; and whether it was resolved.

Project Management and Time Line for the Project

Project start: 1. March 2018
Data retrieval of pre-scanning, pretesting and similar before intervention: 01.02.2018 – 01.06.2018
Recruitment will be continuous until we reach our goal. Recruitment will start in March 2018. Initiation of intervention will also be continuous and start as each participant is scanned and tested. Scanning and testing will be done from March until June, or longer if necessary.

Intervention period: 01.03.2018 – 01.03.2019
The intervention period may vary for the different participants, but not the length, which will last for 12 months. This means that if a participant joins the project on June 1, 2018, the intervention will begin from that date and last for 12 months from that date.

Analysis of brain imaging data: 01.02.2018 to 01.02.2020
Analysis of imaging data will occur on a continuous basis after each participant has been in the scanner and taken the psychological tests. Since this is to be done twice, it is assumed that much time will go for analysis. Statistical analysis comparing data between interventions will not commence before the end of data collection and preparation (database lock) for all participants.

Article writing and publishing of results: 01.02.2020 to 01.02. 2021
During this period, the project team will work on preparing data for publishing, creating guidelines/manuals for interventions that can be used by the municipality and the rest of the country. The intervention material, such as the music, the music program, the exercise program, will be made available for all who wish to use it, both domestic and internationally.

Recruitment end: 01.01.2020
Recruitment will be an ongoing procedure until we get 135 participants or the project run out of time. Hence the last day of recruitment will be a bit before a year is left of the project so that participants still gett the 12 months of intervention and the analysis can be included.

Project end: 01.02.2021
Around the end of the project we wish to send out an evaluation form for participants where we ask if they are still home and whether they have moved into a nursing home. We will also ask if we can contact them two and a half years after intervention (which will be before the project ends) and then again 5 years after end of intervention.

Ethics and Dissemination

Ethical Challenges

The current study is investigating an exposed group, people who have Alzheimer's disease. Even at an early stage in the disease, this group may be considered exposed. For the current study it is important that participants are living at home and the interventions may help them stay home longer. Music and exercise have the health and positive effects on patients with AD, but also generally older people and their caregivers. Nevertheless, there is little high quality research on long-term music and exercise therapy in people with AD. Therefore, this study holds great potential; it is an exploratory study. Little suggests negative effects of song and exercise in people with AD. The literature states that depression and anxiety decreases with music therapy, and well-being increases, but no evidence suggests a stop of disease progression.

The group may not continue to stay home during the intervention, the literature states that deception, anxiety decreases with music therapy, and well-being increases, but it is no evidence that suggests a stop of disease progression. We assume new learning and active use of hippocampus may have positive effects on the brain. If participants move away from home during the period, they will continue to receive the same offers from us. We expect some dropouts from the study, hence we want to test as many patients as possible. If participants no longer wish to do the intervention, but still want to be tested, that will be allowed. If participants wish to do the intervention, but not be tested they will be allowed to do that as well.

fMRI and MR may be experienced as uncomfortable by some participants, but are necessary for the research, however it is not dangerous for the participants.
Retrieval of personal data and neuropsychological tests can also be uncomfortable for some of the participants, but where a computer can be utilized, it will, to limit researcher access and vulnerably of participants.

Music therapy or exercise may not be attractive to everyone. Song has ecological validity in our everyday lives. We humans often meet and sing in different contexts, such as for weddings, funerals, in church, at work and school. We will also ask participants what they prefer of singing,

exercise and no intervention. Although the study will randomise participants, it will indicate why some choose to drop out. The hope is that all participants who are recruited will get enough insight into the study to know that singing lessons, exercise or nothing are the options for participating in the experiment.

The biggest challenge is the control group that will not get any intervention. For the current study, participants are not to be medicated by us, but singing and exercise can be considered a kind of treatment, while the control group is not receiving any treatment. Regular medical treatment that patients usually use is acceptable and they will continue with it during the trial period. That is, the participants get the best beneficial treatment as established in medical research. Music and exercise are two of the best options for non-medical treatment, therefore all groups will receive good treatment. As mentioned in the background section, music and exercise are two of the best options for non-medical treatment, therefore all groups will receive good treatment.

For the current project, co-authorship might become problematic. As there will be three PhD positions on the project, they will all want to be first author on the publications. There are also a limit to how many articles one project can yield. Also as the project has many partners, there might be difficult to detect when which authors should be mentioned in articles or not.

As the project cooperates with Bergen municipality positive findings will be implemented into the health care system. Betterment of health care is a positive implication of the possible findings. However, new music treatment which works may demote other research of being implemented, or research to be done in the field at all. For music may be good medication, it might not be a cure for AD. Also because this is a massive study, which takes time and money, others may not replicate it. Which is why the societal implications of the findings might be negative as well as positive.

Plan for Publication and Utilisation of Research Results

Publishing is scheduled in magazines and peered reviewed papers. We also plan a separate website with material and results after they arrive. If the results are positive, we will, in cooperation with the municipality of Bergen, develop a program for better health for Alzheimer's patients. We will help Bergen Municipality and others to train music therapists and give them access to the music and material we have used. Initially all the material will be given free of charge to all municipalities in Hordaland

References

Bergen, & Kommune. (2010). Trygg på to bein en prosjektrapport ergo og fysioterapitjenesten.

Burke, W. J., Houston, M. J., Boust, S. J., & Roccaforte, W. H. (1989). Use of the Geriatric Depression Scale in dementia of the Alzheimer type. *Journal of the American Geriatrics Society, 37*(9), 856-860. doi:10.1111/j.1532-5415.1989.tb02266.x

Campbell, A. J., Robertson, M. C., Gardner, M. M., Norton, R. N., & Buchner, D. M. (1999). Psychotropic medication withdrawal and a home□based exercise program to prevent falls: a randomized, controlled trial. *Journal of the American Geriatrics Society, 47*(7), 850-853. doi:10.1111/j.1532-5415.1999.tb03843.x

Cuddy, L. L., Duffin, J. M., Gill, S. S., Brown, C. L., Sikka, R., & Vanstone, A. D. (2012). Memory for melodies and lyrics in Alzheimer's disease. *Music Perception: An Interdisciplinary Journal, 29*(5), 479-491. doi:10.1525/mp.2012.29.5.479

Damoiseaux, J. S., Prater, K. E., Miller, B. L., & Greicius, M. D. (2012). Functional connectivity tracks clinical deterioration in Alzheimer's disease. *Neurobiology of aging, 33*(4), 828. e819-828. e830. doi:https://doi.org/10.1016/j.neurobiolaging.2011.06.024

Dassa, A., & Amir, D. (2014). The Role of Singing Familiar Songs in Encouraging Conversation Among People with Middle to Late Stage Alzheimer's Disease. *Journal of Music Therapy, 51*(2), 131-153. doi:10.1093/jmt/thu007

El Haj, M., Clément, S., Fasotti, L., & Allain, P. (2013). Effects of music on autobiographical verbal narration in Alzheimer's disease. *Journal of Neurolinguistics, 26*(6), 691-700.

El Haj, M., Postal, V., & Allain, P. (2012). Music enhances autobiographical memory in mild Alzheimer's disease. *Educational Gerontology, 38*(1), 30-41. doi:https://doi.org/10.1080/03601277.2010.515897

Finke, C., Esfahani, N. E., & Ploner, C. J. (2012). Preservation of musical memory in an amnesic professional cellist. *Current Biology, 22*(15), R591-R592.

Foster, N. A., & Valentine, E. R. (2001). The effect of auditory stimulation on autobiographical recall in dementia. *Experimental aging research, 27*(3), 215-228. doi:https://doi.org/10.1080/036107301300208664

Franke, K., Ziegler, G., Klöppel, S., Gaser, C., & Initiative, A. s. D. N. (2010). Estimating the age of healthy subjects from T1-weighted MRI scans using kernel methods: exploring the influence of various parameters. *NeuroImage, 50*(3), 883-892. doi:https://doi.org/10.1016/j.neuroimage.2010.01.005

Fusar-Poli, L., Bieleninik, Ł., Brondino, N., Chen, X.-J., & Gold, C. (2017). The effect of music therapy on cognitive functions in patients with dementia: a systematic review and meta-analysis. *Aging & mental health*, 1-10. doi:https://doi.org/10.1080/13607863.2017.1348474

Guetin, S., Portet, F., Picot, M.-C., Pommié, C., Messaoudi, M., Djabelkir, L., . . . Touchon, J. (2009). Effect of music therapy on anxiety and depression in patients with Alzheimer's type dementia: randomised, controlled study. *Dementia and geriatric cognitive disorders, 28*(1), 36-46.

Guralnik JM, S. E., Ferrucci L, Glynn RJ, Berkman LF, Blazer DG, Scherr PA, Wallace RB. (1994). A short physical performance battery assessing lower extremity function: association with self-reported disability and prediction of mortality and nursing home admission. *J Gerontol, 49*(2), 85-94.

Helbostad, J. L., Leirfall, S., Moe-Nilssen, R., & Sletvold, O. (2007). Physical fatigue affects gait characteristics in older persons. *The Journals of Gerontology Series A: Biological Sciences and Medical Sciences, 62*(9), 1010-1015. doi:https://doi.org/10.1093/gerona/62.9.1010

Hsieh, S., Hornberger, M., Piguet, O., & Hodges, J. R. (2011). Neural basis of music knowledge: evidence from the dementias. *Brain, 134*(9), 2523-2534. doi:https://doi.org/10.1093/brain/awr190

Hulme, C., Wright, J., Crocker, T., Oluboyede, Y., & House, A. (2010). Non□pharmacological approaches for dementia that informal carers might try or access: a systematic review. *International journal of geriatric psychiatry, 25*(7), 756-763. doi:10.1002/gps.2429

Irish, M., Cunningham, C. J., Walsh, J. B., Coakley, D., Lawlor, B. A., Robertson, I. H., & Coen, R. F. (2006). Investigating the enhancing effect of music on autobiographical memory in mild Alzheimer's disease. *Dementia and geriatric cognitive disorders, 22*(1), 108-120. doi:https://doi.org/10.1159/000093487

Jacobsen, J.-H., Stelzer, J., Fritz, T. H., Chételat, G., La Joie, R., & Turner, R. (2015). Why musical memory can be preserved in advanced Alzheimer's disease. *Brain, 138*(8), 2438-2450. doi:https://doi.org/10.1093/brain/awv135

Jäncke, L. (2009). The plastic human brain. *Restorative neurology and neuroscience, 27*(5), 521-538. doi:10.3233/RNN-2009-0519

Kalapotharakos, V. I., Michalopoulos, M., Tokmakidis, S. P., Godolias, G., & Gourgoulis, V. (2005). Effects of a heavy and a moderate resistance training on functional performance in older adults. *Journal of strength and conditioning research, 19*(3), 652.

Koelsch, S. (2014). Brain correlates of music-evoked emotions. *Nature reviews Neuroscience, 15*(3), 170-180. doi:10.1038/nrn3666

Kommune, B. (2010). Trygg på to bein en prosjektrapport ergo og fysioterapitjenesten.

Liu, C. j., & Latham, N. K. (2009). Progressive resistance strength training for improving physical function in older adults. *The Cochrane Library*. doi:10.1002/14651858.CD002759.pub2

McDermott, O., Crellin, N., Ridder, H. M., & Orrell, M. (2013). Music therapy in dementia: a narrative synthesis systematic review. *International journal of geriatric psychiatry, 28*(8), 781-794. doi:10.1002/gps.3895

Moussard, A., Bigand, E., Belleville, S., & Peretz, I. (2012). Music as an aid to learn new verbal information in Alzheimer's disease. *Music Perception: An Interdisciplinary Journal, 29*(5), 521-531. doi:10.1525/mp.2012.29.5.521

Münte, T. F., Altenmüller, E., & Jäncke, L. (2002). The musician's brain as a model of neuroplasticity. *Nature reviews Neuroscience, 3*(6), 473. doi:10.1038/nrn843

Nir, T. M., Jahanshad, N., Villalon-Reina, J. E., Toga, A. W., Jack, C. R., Weiner, M. W., . . . Initiative, A. s. D. N. (2013). Effectiveness of regional DTI measures in distinguishing Alzheimer's disease, MCI, and normal aging. *NeuroImage: clinical, 3*, 180-195. doi:https://doi.org/10.1016/j.nicl.2013.07.006

Oostendorp, J. C., & Montel, S. R. (2014). Singing Can Enhance Episodic Memory Functioning in Elderly People with Alzheimer's Disease. *Journal of the American Geriatrics Society, 62*(5), 982-983. doi:10.1111/jgs.1273

Osman, S. E., Tischler, V., & Schneider, J. (2016). 'Singing for the Brain': A qualitative study exploring the health and well-being benefits of singing for people with dementia and their carers. *Dementia, 15*(6), 1326-1339. doi:10.1177/1471301214556291

Pantev, C., & Herholz, S. C. (2011). Plasticity of the human auditory cortex related to musical training. *Neuroscience & Biobehavioral Reviews, 35*(10), 2140-2154. doi:https://doi.org/10.1016/j.neubiorev.2011.06.010

Satoh, Yuba, Tabei, Okubo, Kida, Sakuma, & Tomimoto. (2015). Music Therapy Using Singing Training Improves Psychomotor Speed in Patients with Alzheimer's Disease: A Neuropsychological and fMRI Study. *Dementia, 5*, 296-308. doi:10.1159/000436960

Selkoe, D. J. (2001). Alzheimer9s Disease: Genes, Proteins, and Therapy. *Physiological reviews, 81*(2), 741-766. doi:doi:10.1038/399a023

Skelton, D., Dinan, S., Campbell, M., & Rutherford, O. (2005). Tailored group exercise (Falls Management Exercise—FaME) reduces falls in community-dwelling older frequent fallers (an RCT). *Age and ageing, 34*(6), 636-639. doi:https://doi.org/10.1093/ageing/afi174

Skingley, A., Martin, A., & Clift, S. (2016). The contribution of community singing groups to the well-being of older people: Participant perspectives from the United Kingdom. *Journal of Applied Gerontology, 35*(12), 1302-1324. doi:https://doi.org/10.1177/0733464815577141

Särkämö, T., Tervaniemi, M., Laitinen, S., Forsblom, A., Soinila, S., Mikkonen, M., . . . Laine, M. (2008). Music listening enhances cognitive recovery and mood after middle cerebral artery stroke. *Brain, 131*(3), 866-876. doi:https://doi.org/10.1093/brain/awn013

Tarr, B., Launay, J., & Dunbar, R. I. (2014). Music and social bonding: "self-other" merging and neurohormonal mechanisms. *Front Psychol, 5*(1096). doi:10.3389/fpsyg.2014.01096
Ueda, T., Suzukamo, Y., Sato, M., & Izumi, S.-I. (2013). Effects of music therapy on behavioral and psychological symptoms of dementia: a systematic review and meta-analysis. *Ageing research reviews, 12*(2), 628-641. doi:https://doi.org/10.1016/j.arr.2013.02.003
van der Steen, J. T., van Soest□Poortvliet, M. C., van der Wouden, J. C., Bruinsma, M. S., Scholten, R. J., & Vink, A. C. (2017). Music□based therapeutic interventions for people with dementia. *The Cochrane Library*. doi:10.1002/14651858.CD003477.pub3
Vanstone, A. D., Sikka, R., Tangness, L., Sham, R., Garcia, A., & Cuddy, L. L. (2012). Episodic and semantic memory for melodies in Alzheimer's disease. *Music Perception: An Interdisciplinary Journal, 29*(5), 501-507. doi:10.1525/mp.2012.29.5.501
Vickhoff, B., Malmgren, H., Astrom, R., Nyberg, G., Ekstrom, S. R., Engwall, M., & Jornsten, R. (2013). Music structure determines heart rate variability of singers. *Front Psychol, 4*(334). doi:10.3389/fpsyg.2013.00334
Vink, A. C., Bruinsma, M. S., & Scholten, R. J. (2003). Music therapy for people with dementia. *The Cochrane Library*. doi:10.1002/14651858.CD003477.pub2
Wall, M., & Duffy, A. (2010). The effects of music therapy for older people with dementia. *British journal of nursing, 19*(2), 108-113. doi:https://doi.org/10.12968/bjon.2010.19.2.46295

Appendix

Appendix A Singing lessons

Sangundervisning/sangtimer

Tid: 60 min

Oppvarming – 15 minutter

Tøy og bøy

a. Strekke armene mot taket, hold pusten, ta armene ned – slipp pusten ut (Høge fjell og djupe daler).
b. Rulle på skuldrane, begge veier (Anne Malene)
c. Strekke fingrane / rotere handledda (Mamma, får eg lov å gå på kino)
d. Strekke beina (Nidelven)
e. Trampe takt (O Marie, Pengegaloppen, Bedre og bedre dag for dag, Davy Crockett, Napoleon med sin hær)
f. Hæl / tå (Mellom bakker og berg 1.vers)
g. Ro- øvelser (En sjømann elsker havets våg)

Mia o Maria Mia o Maria

Vioala viola viola viola vi

Ja det var bra ja, bra ja, ja det var bra

Lekesynging – 20 minutter

Bro bro brille
Alle fugler små de er
Jeg gikk en tur på stien
Pål sine høner
Ro ro ro din båt
Fader jacob på flere språk

Korsang: - 25 min

Månedens sang

Lytting: 5 min

Sangliste

Appendix B Exercise Lessons

Treningsprogram
Basert på trygg på to bein

Tid: 60 min

Oppvarming:

Velkommen, opprop

Sitte på stol oppvarming:
Armer – strekk mot taket
Skuldre – Hev, senke, rulle

Stå bak stolen:
Sving og sikt, skitak ”Ski-tak”, Sving side til side, Sving side – heilt rundt
Gå på staden, Med armsving
Side steg, Ett steg sideveis, fram og tilbake.

Gange bak stolradene:
Gå framover
Gå bakover – bytt vei
Gå sidesteg – bytt vei

Hoveddel:

Styrkeøvelser:
Stå bak stolen:
Opp på tå 10x3 repetisjoner
Opp på hæl 10x3 rep
Abduksjon i hofter
Opp på tå, ta side steg 10x3 rep.
Instruer kvar enkelt i å stå stabilt på en fot, evt. start med tyngdeoverføring - 10x3 rep
Knebøy - 10x3 rep

Stå med siden intill stolen:
Knebøy med framfall 10x3, skift bein
Høge kneløft 10x3, skift bein
Knebøy på en fot - 10x3 rep, - skift bein - Støtte på stol. Skift bein 10x3

Sitte på stolen:
Reise seg opp- sitt ned – uten støtte på stol 10 x 3
Reise seg opp- sitt halvveis ned – reis opp – start m 5, så 7 også 10 (3 rep)
Reise seg opp- sitt halvveis ned og hald stillingen i 3 sekund- reis opp - Støtte på låra i starten, gradvis uten støtte og lenger hald.
Reise seg opp- sitt nesten ned- raskt opp igjen - ”Tegnestift på stolen” 10 rep, auke til 10x3
Reise seg opp på matte, leg matte intil stolen.

Gå med høye kneløft rundt i rommet

Balanseøvelser:
sitte på stolen – reise seg, gå rundt stolen rolig.
Reise opp, sidesteg til neste stol, sitt ned. - gjenta rundt hele sirkel
Reise opp og sitt ned og bytt beina.

Stå bak stolen:
Stå med samla bein, Med åpne øyne
Stå med samla bein - Med lukkede øyne- tell til 5 i starten.
Stå i gangstilling -Med sida mot stolen. Tyngdeoverføring fram/tilbake
Stå på en fot -Støtte på stol i starten. Skifte bein.
Snu rundt 360 grader, rolig og skift retning

To sidesteg til høyre - opp på tå - to sidesteg til venstre
Slalåm mellom stolene – gå en runde
Gå rolig. Skift retning - Gjenta 5 ganger
Gå med vekselvis -"musesteg" og lange steg framover

Gå og bære - glas/pappkrus med vatn
Gå over stepkasse - Støtte ved behov.
Gå på airex matte med små erteposer el skjult under.
Ballkast ved gange Personstøtte ved behov - Støtte ved behov
Terapeut kaster ball til gående deltakere – tilfeldig rekkefølge.

Stå ved vegg eller ribbevegg - Steppe Frontsteg, sidesteg, steg over step
I gangbane Stående med stol framfor
Stående med stol bak
Stående med sida inntil stol
Inntil vegg - Gå på strek
Stå på en fot - Tape strek eller legg treningsstrikk på golvet, gå framover, bakover, sideveis reduser støtte etter kvart. Skift bein
Ballkast med terapeut - Varier med sidesteg mellom kvart mottak.
Stå i tandemstilling Gå på matte(r) Begynn gjerne i gangstilling og reduser understøttelsesflate etterkvart
Gå i ulike retninger, evt. sikre med stoler
Inntil vegg eller mellom to stoler
Sitte på stol Stå på matte(r) Gangstilling, beina sammen, tandemstilling.
Oppreising Ulike varianter av oppreising som over.

Leker som mus og katt osv.

Nedvarming:

Tøye ut, nedvarming – gjør det som passer for gruppa, sitt på stol. Slappe av, puste ut.

Appendix C Music used for the brain scans

Music for the scanner, deltakerne får velge en av følgende sjangere:

1. Klassisk stykke - Praetorius Six Dances from Terpsichore

https://www.youtube.com/watch?v=kmHR6GWQOWU

2. Rock – Wild Melodic Metal | Guitar Backing Track Jam

https://www.youtube.com/watch?v=R0jwsidG4wU

3. World - PAN FLUTE PERU - INKA TRAIL - CAMINO INCA

https://www.youtube.com/watch?v=48FZKfcRauM

4. Jazz –Modern jazz gypsy

https://www.youtube.com/watch?v=YkrxvqGbqgk

5. Pop – 80s Pop Funk Backing Track (Dm) | 110 bpm - MegaBackingTracks

https://www.youtube.com/watch?v=tD8AtHHvzJs

6. Folk - samme som World

NB: Alle sangene har blitt behandlet i Audiocity og endret til 74 decibel (dB) og normalisert for lydvariation. Alle sangene er også blitt kuttet og satt sammen til å vare 8 minutter. Selve skanningen varer 7.14 minutter.

Appendix D Testing protocol

<u>Forskningsprotokoll Tester</u>

Testing foregår inne på rommet ved siden av fMRI rommet. Hente lånekort i U3 skanneren. Sett deltakeren i den svarte sofaen, og sett deg i den svarte stolen som vende mot sofa.

"Hei, kjekt at du er med på prosjektet. I dag skal vi gjennom en del tester og spørreskjema og lignende. Først så trenger jeg at du skriver under på denne erklæringen om informert samtykke. Les den først og si ifra hvis det er noe som er uklart."

La deltakeren lese gjennom informert samtykkeskjema, dette tar ofte litt tid. Gjør klar alle skjemane dere skal gjennom mens du venter. Signer så samtykke skjema du også, med navn, dato og rolle på prosjektet.

1. Spørreskjema

"Jeg vil du skal fylle ut dette skjema, bare spør hvis det er noe som er utydelig,"

Gi deltakeren blyant så de kan fylle ut, ikke overvåk dem. Hvis det er åpenbart at de står fast, hjelp.

2. Ord læringsliste

"Flott, du gjør det kjempe bra. Nå skal vi se på litt ordlæring og husking av en ordliste. Først får du en ordliste med ti hverdagslige ord, jeg viser deg arket også vill jeg at du skal lese høyt hva som står der, etterpå vil jeg at du skal si alle ordene du nettop leste."

Vis deltakeren ordene i riktig rekkefølge med to sekunders intervall, rekkefølge 1. Hvis deltaker ikke kan lese et ord, les det til ham og skriv det ned for det tilsvarende ordet i kolonnen «Kan ikke lese».

REKKEFØLGE 1:

1. Smør
2. Dårlig
3. Strand
4. Brev
5. Dronning
6. Hytte
7. Stang
8. Kart
9. Gress
10. Motor

«Flott, nå kan du si hvilken ord du nettop leste?»

Gi deltakeren 90 sekunder, rekkefølgen spiller ingen rolle. Skriv ned hvor mange ord deltakeren klarer. Gjenta prosessen, sorter ordene i rekkefølge 2 før du holder dem opp igjen.

«Flott, da kan du lese opp ordene igjen.»

REKKEFØLGE 2:

1. Kart
2. Hytte
3. Smør
4. Strand
5. Motor
6. Dårlig
7. Dronning
8. Brev
9. Stang
10. Gress

«Bra, kan du gjenta ordene du nettop leste?»

Skriv ned og gjenta prosess for en tredje gang, to sekunders intervall. Sorter kortene i rekkefølge 3.

REKKEFØLGE 3:

1. Dronning
2. Gress
3. Dårlig
4. Hytte
5. Stang
6. Strand
7. Smør
8. Motor
9. Kart
10. Brev

3. ADL

"Oki, supert. Nå skal jeg spørre deg litt om hva du gjør i hverdagen,"

Still spørsmålene og se om svaret passer i noen av boksene, hvis ikke kan du grave litt for å få et alternativ som passer i boksene. Gå tilbake og bruk ordliste to for gjenkjennelse og gjenkallelse.

2.2 Gjenkallelse ordlæringstest

"For noen minutter siden ba jeg deg om å lære en liste med 10 ord. Nå vil jeg at du skal huske disse ordene"

"Nå skal jeg gi den masse ord skrevet på kort. Noen av disse ordene har du allerede sett, og noen av ordene er nye. Jeg vil at du skal si "Ja" hvis order jeg viser deg er et du har allerede sett før"

Sorter inn de nye ordene og bruk rekkefølge 4.

REKKEFØLGE 4:

1. Kirke
2. Kaffe
3. **Smør**
4. Dollar
5. **Dårlig**
6. **Strand**
7. Fem
8. **Brev**
9. Hotel
10. Berg
11. **Dronning**
12. **Hytte**
13. Tøffel
14. **Stang**
15. Landsby
16. Band
17. **Kart**
18. Hær
19. **Gress**
20. **Motor**

4. GDS

"Kjempe bra. Nå skal jeg spørre deg litt om humøret ditt og hvordan du føler deg til vanlig. Jeg vil at du skal fylle ut denne listen,"

Gi deltakeren GDS og la de svare selv, med blyant som de allerede har. Hjelp ved behov.

5. MMSE

"Du er kjempe flink. Går det bra? Trenger du en pause?"

Gi pause på 5 til 10 minutter hvis de trenger det.

"Oki, la oss gå videre, nå skal vi teste litt forskjellige kunnskaper,"

Gi deltaker blyant og viskelær. Utfør og følg instruksene til MMSE på selve testen.

6. SPPB

"Veldig bra, nå skal vi få litt fysisk aktivitet. La oss nå begynne kartleggingen. Nå vil jeg at du skal prøve å innta ulike stillinger. Jeg vil først beskrive og vise hver stilling for deg. Så vil jeg at du skal prøve å gjøre det samme. Du skal ikke gjøre noe du føler er utrygt eller noe du ikke klarer. Har du noen spørsmål før vi starter?"

Husk at du skal bruke stoppeklokke. Gjerne si ifra om at det er det du gjør på mobilen din, sånn at de ikke blir fornærmet.

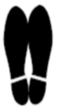

1) Begynn med stående stilling, samlede føtter.

«Nå vil jeg vise deg stillingen» *demonstrer* «Jeg vil at du skal forsøke å stå med føttene samlet, inntil hverandre i ca 10 sekunder. Du kan bruke armene, bøye knærne eller bevege kroppen for å holde balansen, men prøv å ikke flytte på føttene. Prøv å holde stillingen til jeg ber deg stoppe»

Husk stoppeklokke og scoringsark. Stå ved siden av deltakeren for å hjelpe han/henne inn i stillingen. Gi akkurat nok støtte til deltakerens arm for å unngå at personen mister balansen. Når deltakeren er i stillingen spør du

«Er du klar?» *Slipp takket og start tidtakingen idet du sier:* «Klar, start»

Stopp stoppeklokken og si «Stopp» *etter 10 sekunder eller hvis deltakeren flytter føttene og forlater stillingen eller griper tak i armen din.*

Hvis deltakeren ikke klarer å holde stillingen i 10 sekunder noter resultatet og gå videre til ganghastighet, dropp altså de andre stående øvelsene.

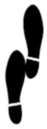

2) Stående stilling - semi-tandem.

«Nå skal jeg vise deg den andre stillingen» *demonstrer* «Nå vil jeg at du skal forsøkte å stå med ved siden av hælen på den ene foten og inntil stortåen på den andre foten i ca 10 sekunder. Du kan velge hvilken fot du har først, det som er naturlig for deg. Du kan bruke armene, bøye knærne eller bevege kroppen for å holde balansen, men prøv å ikke flytte på føttene. Prøv å holde stillingen helt til jeg ber deg stoppe»

Stå ved siden av deltageren for å hjelpe han/henne inn i semi-tandem stillingen. Gi akkurat nok støtte til deltagerens arm for å unngå at hun/han ikke mister balansen. Når deltakerens føtter står samlet, spør du:

«Er du klar?» *Slipp takket og start tidtakingen idet du sier:* «Klar, start»

Stopp stoppeklokken og si «Stopp» *etter 10 sekunder eller hvis deltageren flytter føttene og forlater stillingen eller griper tak i armen din.*

Hvis deltakeren ikke klarer å holde stillingen i 10 sekunder noter resultatet og gå videre til ganghastighet, dropp altså de andre stående øvelsene.

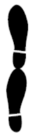

3) Stående stilling - tandem

«Nå skal jeg vise deg den tredje stillingen» *demonstrer* «Nå vil jeg at du skal forsøkte å stå med hælen på den ene foten og inntil tærne på den andre foten i ca 10 sekunder. Du kan velge hvilken fot du har først, det som er naturlig for deg. Du kan bruke armene, bøye knærne eller bevege kroppen for å holde balansen, men prøv å ikke flytte på føttene. Prøv å holde stillingen helt til jeg ber deg stoppe»

Stå ved siden av deltageren for å hjelpe han/henne inn i semi-tandem stillingen. Gi akkurat nok støtte til deltagerens arm for å unngå at hun/han ikke mister balansen. Når deltakerens føtter står samlet, spør du:

«Er du klar?» *Slipp takket og start tidtakingen idet du sier:* «Klar, start»

Stopp stoppeklokken og si «Stopp» *etter 10 sekunder eller hvis deltageren flytter føttene og forlater stillingen eller griper tak i armen din.*

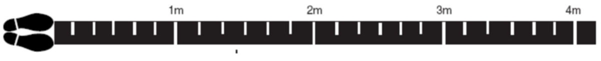

4m Gangtest

«Nå skal jeg observere hvordan du vanligvis går. Hvis du bruker stokk eller andre ganghjelpemidler og føler du trenger det for å gå en kort distanse kan du bruke det.»

A) *Første del av ganghastighet*

«Denne distansen skal du gå. Jeg vil at du skal gå til den andre enden, i din vanlige hastighet, som om du gikk nedover gaten til butikken.»

Demonstrer øvelsen for deltageren. Bruk oppmarkert rute i rommet. Det er målt til 4 meter.

«Gå hele lengden, over og forbi teip-markeringen før du stopper. Jeg kommer til å gå sammen med deg. Føler du at dette er trygt?»

La deltageren stå med begge føttene inntil startlinjen.

«Når jeg sier du skal starte sier jeg: Klar start.» *Når deltageren bekrefter å ha forstått instruksjonen si:*
«Klar, Start»

Start tidtakingen idet deltageren begynner å gå. Gå bak og til siden for deltageren. Stopp tidtakingen når en av deltagerens føtter er helt over mållinjen.

B) Andre test av ganghastighet

«Nå vil jeg at du skal gjøre det samme en gang til. Husk å gå i din vanlige hastighet, og gå helt over og forbi teip-markeringen.»

La deltageren stå med begge føttene inntil startlinjen.

«Når jeg sier du skal starte sier jeg: Klar start.» Når deltageren bekrefter å ha forstått instruksjonen si:
«Klar, Start»
Start tidtakingen idet deltageren begynner å gå. Gå bak og til siden for deltageren. Stopp tidtakingen når en av deltagerens føtter er helt over mållinjen.

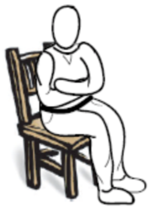

Reise seg test

1) Reise seg fra stol én gang

«Dette er den siste øvelsen. Er det trygt for deg å reise deg opp fra stolen uten å bruke armene? Denne testen måler styrken i beina dine.»

Demonstrer og forklar øvelsen. «Først kryss armene over brystet, og sitt slik at føttene er plassert på gulvet: så reiser du deg opp, behold armene i kryss over brystet. Nå vil jeg at du skal prøve å reise deg opp med armene i kryss over brystet.»

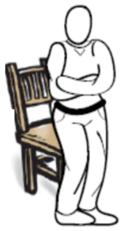

Noter resultatet, stolen er 44 cm høy. Hvis deltakeren ikke klarer å reise seg uten å bruke armene si:
«Ok, prøv å reise deg med å bruke armene»
Dette avslutter testen, noter resultatet.

2) *Reise/ sette seg x5*

«Tror du det vil være trygt for deg å reise deg opp fra stolen fem ganger uten å bruke armene?»

Demonstrer og forklar øvelsen: «Nå vil jeg at du skal reise deg helt opp, så RASKT du kan fem ganger, uten stopp. Etter at du har reist deg hver gang, sett deg ned og reis deg opp igjen. Behold armene i kryss over brystet. Jeg tar tiden med en stoppeklokke.»

Når deltakeren sitter på riktig måte, si: «Klar? Reis deg» *og start tidtakingen. Tell høyt hver gang deltageren reiser seg, opp til fem ganger. Stopp om deltageren blir sliten eller tungpustet av å reise seg fra stolen flere ganger. Stopp stoppeklokka når han/hun har reist seg helt opp den femte gangen.*
Stopp også hvis deltakeren bruker armene, etter 1 minutt ikke har fullført 5 repetisjoner. Hvis du er bekymret for deltakerens sikkerhet.

Hvis deltakeren er sliten og stopper før fem repetisjoner spør du: «Kan du fortsette?» *For å bekrefte dette. Hvis deltageren sier* «Ja», *forsett tidtakingen. Hvis deltageren sier* «Nei,» *stopp og nullstill stoppeklokken.*

7. STROOP

"Kjempe flott, nå skal du få gjøre noen tester på denne datamaskinen."

Gi deltakeren pause mens du setter opp datamaskinen og går inn på STROOP programmet, søk i søkefeltet og åpne psychopy filen, dette tar noen minutter, trykk på RUN (grønne knapp og gi data til deltakeren. Forklar ekstra, dette er vanskelig å forstå.)

8. Finger Tapping Test

“Den testen er veldig vanskelig, meningen man skal gjøre feil, men du var flink. Nå skal du bare trykke på mellomromstasten masse”.

Finn FTT, ved å søke på FTT eventuelt åpne previous experiments i psychopy. Klikk på run og gi til deltakeren.

9. PROMS

“Nå er vi snart ferdig. Du skal nå få en test som skal se litt på musikk evnene dine,”

Søk på internett explorer (finn favoritter og trykk på PROMS linken. Hvis internett ikke er koblet til så går du på wifi knappen og trykker på koble til eudoram.
Bruk deltakernummer og ikke det som står på introduksjonssiden for dem. Fyll inn første side for dem som et intervju.

Finn fram test headsettet og la deltakeren ta dem på. Og hjelp dem med å justere lyd og starte testen.

10. FMRI

“Kjempe flott, da er vi ferdig, du har vært en sikkelig helt. Nå skal du snart få gå inn i skanneren hvor vi skal se på hjernen din. Men først skal du få litt pause, trenger du å gå på do? Spise osv?”

Vise dem hvor kantinen og Narvesen er hvis de er sulten, og toalettet er rett nede i gangen. Når dere er klare og det er fMRI tiden deres går dere rett over gangen og bort til skanneren. Derfra følg egen protokoll og introduser deltakeren til radiografene.

GODT JOBBA!

Appendix E fMRI Protokoll

FMRI protokoll

Muntlige instrukser før skanning begynner:

Nå skal du inn i fMRI maskinen. Det er for at vi skal kunne se hvordan hjernen din ser ut. Det hele tar ca 30 minutter. Har du lyst til å høre på musikk mens du er der inne? Hva slag type musikk liker du?

Vi har klassisk, rock, pop, world, folk og jazz musikk.

Flott! Det er viktig at du slapper helt av og har øynene luket. Jeg setter på et bildeshow med naturbilder; men du trenger ikke se på det. Og når vi sier at du må lukke øynene er det veldig viktig at du gjør det.

(Gi deltakeren og ID nummer til radiografene, de går gjennom sjekkliste og låser inn eiendeler osv)

Skru på datamskinen, eller få radiografene til å hjelpe deg å logge på. Gå inn på datamaskinen - paradigmer (R) - ALMUTH og velg musikksjanger. Sett på power point presentasjonen og dra den over på den andre skjermen. Pass på at skjermen er på ved å trykke på M2 på den svarte boksen. Sjekk at den hvite boksen har fMRI data knappen på 80 % volum. Og at master volumet er stilt inn på EMO lydnivå. Og pass på at Windows Media Player er på fult.

Nå starter opptakene. (sier radiografene, de tar over snakking fra nå av)

LOC
Asset cal 33d
SAG
DTI

Mens disse går, skriv inn all data for deltaker-nummer, dato, ID, Musikk, Sjanger, fMRI og eventuelle merknader underveis. Husk å skrive hvem som samler inn data også, (altså hvem du er). Bruk 1. Side som mal.

Nå er det klart for fMRI. Skriv hvilken som begynte med, musikk eller ikke musikk. Stopp den andre musikken og lysbildene. Skru av skjermen. (svart boks, trykk på M1)

Hei, nå kommer det to opptak hvor du skal ligge og tenke på det du vil. Her er det veldig viktig at du har øynene lukket. Det ene opptaket du skal høre på musikk, men ikke i det andre.

Sett på den andre filen i mappen med musikk merket MR også f. Eks jazzsang. Alle sangene er merket med «Sang» på slutten av sjangeren. Sett på musikken når radiografene setter på opptaket. Alle sangene er lengre enn opptaket, så ikke vær bekymret for å gå tom for tid. Husk at annenhver gang skal musikken først og annenhver gang sist. Sjekk hva som ble gjort sist ved å bla i fMRI boken. Når opptaket er ferdig stanser du musikken.

Da var du ferdig. Ta med deltakeren ut og si at de gjorde en god jobb, uavhengig om de gjorde det eller ikke. Deretter følg dem dit de skal nå. Husk du er ansvarlig.

Godt jobba!
